# Renewable DNA Proportional-Integral Controller with Photoresponsive Molecules

**DOI:** 10.3390/mi13020193

**Published:** 2022-01-26

**Authors:** Masaaki Tamba, Keiji Murayama, Hiroyuki Asanuma, Takashi Nakakuki

**Affiliations:** 1 Department of Systems Design and Informatics, Kyushu Institute of Technology, Iizuka 8208502, Japan; tamba.masaaki231@mail.kyutech.jp; 2 Department of Biomolecular Engineering, Graduate School of Engineering, Nagoya University, Nagoya 4648603, Japan; murayama@chembio.nagoya-u.ac.jp (K.M.); asanuma@chembio.nagoya-u.ac.jp (H.A.); 3 Department of Intelligent and Control Systems, Kyushu Institute of Technology, Iizuka 8208502, Japan

**Keywords:** dynamic DNA nanotechnology, strand displacement reaction, proportional-integral controller, photoresponsive molecules, renewable circuit

## Abstract

A molecular robot is an intelligent molecular system. A typical control problem of molecular robots is to maintain the concentration of a specific DNA strand at the desired level, which is typically attained by a molecular feedback control mechanism. A molecular feedback system can be constructed in a bottom-up method by transforming a nonlinear chemical reaction system into a pseudo-linear system. This method enables the implementation of a molecular proportional-integral (PI) controller on a DNA reaction system. However, a DNA reaction system is driven by fuel DNA strand consumption, and without a sufficient amount of fuel strands, the molecular PI controller cannot perform normal operations as a concentration regulator. In this study, we developed a design method for a molecular PI control system to regenerate fuel strands by introducing photoresponsive reaction control. To this end, we employed a photoresponsive molecule, azobenzene, to guide the reaction direction forward or backward using light irradiation. We validated our renewable design of the PI controller by numerical simulations based on the reaction kinetics. We also confirmed the proof-of-principle of our renewable design by conducting experiments using a basic DNA circuit.

## 1. Introduction

In recent years, remarkable progress has been made in molecular computing, which has emerged as a fusion field between computer science, chemistry, and biology. The design of information processing through chemical reactions of biomolecules is a major part of molecular computing; in particular, dynamic DNA nanotechnology, which designs a variety of logical and operational circuits in DNA reaction systems, has been in the spotlight [1,2,3,4,5,6,7,8]. These previous studies demonstrated that it is possible to make various molecular machines intelligent by using functional circuits implemented in molecular reaction systems.

Molecular robots are autonomous mobile systems composed of biomolecules. In a typical design example [9], a body made of phospholipid bilayers, sensors that receive external signals, actuators that generate forces for movement and deformation, and information processing that controls actuators based on external signals obtained from sensors are present. For example, in the ameba-type molecular robot [10], the momentum of the actuator is regulated by the concentration of DNA strands involved in the microtubule-kinesin interaction. The controller design problem of molecular robots is to maintain the concentration of a specific DNA strand at the desired level, which is called a regulator design problem in control engineering [11].

One of the most promising methods for designing a molecular regulation circuit is the rational design based on pseudo-linearization, where three basic reactions (catalysis, degradation, and annihilation) are employed as a component circuit to create a high-dimensional nonlinear system [12]. Utilizing the pseudo-linearization method enables us to design a practical controller based on the linear control theory, such as proportional-integral (PI) controllers [12,13,14] and sliding mode controllers [15]. The computational power of the DNA circuits is severely limited by the finiteness of fuel DNA strands; since the regulation circuit is driven by continuous consumption of fuel strands, the control performance of regulation gradually declines over time. This phenomenon is known as the “finite-time regulation” of DNA-based regulators [16]. The PI and sliding mode controllers also suffer from the same problem and can function normally only within a finite period as long as a sufficient amount of fuel strands remains in the reaction field [17].

One possible strategy to overcome the finite-time regulation problem is to refuel the strands using additional mechanisms, such as buffering or recycling [18,19]. However, for an encapsulated reaction field closed by a liposome, such as molecular robots, it is technically difficult to supply the fuel strand from the outside of the molecular robot. Therefore, in general, for a closed reaction field, another methodology is needed to recover the fuel strand concentrations to their initial concentrations. This can be considered as a problem of the renewability of DNA circuits. In this context, it is necessary to establish a method to make regulation circuits “renewable”.

One of the methodologies to make a DNA circuit renewable is by adding new DNA strands during the operation of the circuit so that the fuel strands consumed in the forward reactions can be regenerated [8,20,21]. This methodology cannot be applied to molecular robots because it has the same problem as the buffering and recycling methods described above. The second methodology is to utilize photoresponsive reaction control in which the DNA strands are modified with the photoresponsive molecule azobenzene to alter the forward and reverse reaction rates depending on light irradiation [22,23]. Azobenzene can destabilize the double-stranded structure of DNA strands under ultraviolet (UV) irradiation [22]. Although it is possible, in principle, to design a regenerable PI controller using the controlled photoresponse method, it has not been attempted to date.

In this study, we propose a new design to make the biomolecule-implemented PI controller developed in [14] (hereinafter referred to as DNA-PI controller) renewable. The innovative contributions of our study are as follows: (1) using the renewable DNA-PI controller as a benchmark example, we showed that by making the individual modules renewable, the entire DNA circuit constructed by combining them can also become renewable, and (2) we performed experimental validation of the renewable design using azobenzene to modify DNA circuits. For this purpose, we adopted a renewable design based on the photoresponsive molecule, azobenzene, proposed by Song et al. [23]. In our previous work [24], we developed a design to make the three basic circuits (catalysis, degradation, and annihilation) of the pseudo-linearization method renewable. In this study, we constructed a renewable DNA-PI controller by applying our renewable pseudo-linearization method to a conventional PI controller. We validated our design via numerical simulations based on reaction kinetics. In addition, we experimentally achieved the proof-of-principle of our renewable design, where we evaluated the degradation circuit of the pseudo-linearization method.

## 2. Materials and Methods

### 2.1. Simulation Conditions

All the simulations were performed using MATLAB (The Mathworks, Inc., Natick, MA, USA). The reaction rates used in the simulations were calculated based on [25] and are listed in Table 1.

### 2.2. Experimental Environment

All DNA sequences used in our experiments are listed in Table 2. Synthesis and azobenzene/fluorescence (Cy5 and BHQ) modifications were obtained from Hokkaido System Science Co., Ltd., Tokyo, Japan. The complex of each gate was generated by annealing a mixture of each DNA from 90 ${}^{\circ }$C to 20 ${}^{\circ }$C at 1 ${}^{\circ }$C/min in a thermal cycler (ASTEC Co., Ltd., Fukuoka, Japan: GeneAtlas 325). DNA was diluted in 1×TE buffer (Nippon Gene Co., Ltd., Toyama, Japan) with 12.5 mM or 6.25 mM ${\mathrm{MgCl}}_{2}$. The signal intensity was measured using a fluorescence spectrophotometer (FP8300; JASCO Corporation, Tokyo, Japan). The measurement conditions were as follows: excitation wavelength of 643 nm, fluorescence wavelength of 667 nm, a bandwidth of 5 nm to Em 5 nm, a data interval of 60 s, and sensitivity was kept at medium. Light irradiation was performed by connecting a digital power supply of PD3-5024-4-EI (CCS Inc., Kyoto, Japan) to an illuminator of HLV2-24UV2-365 (CCS Inc., Kyoto, Japan: 365 nm) for the ultraviolet line and HLV3-22BL-2 (CCS Inc., Kyoto, Japan: wavelength 465 nm) for the blue line.

## 3. Preliminaries

In this section, we briefly summarize the original design of the molecular PI controller [12], which was modified to acquire renewability in this study.

### 3.1. Three Basic Reaction Mechanisms

The signal in a DNA circuit is expressed in terms of molecular concentration. Since concentration is a non-negative value, the biomolecular circuit cannot handle negative signals. To alleviate this restriction, the dual-rail representation can be employed to express the concentration as a negative value. Let a real number u∈R be theoretically represented by the following equation:

(1) $$u=u^{+}-u^{-},$$

where $u^{+}$ and $u^{-}$ are the concentrations of the chemical species $U^{+}$ and $U^{-}$, respectively. For example, the negative value of u=−3 can be expressed as $u^{+}=0$ and $u^{-}=3$. Based on the dual-rail representation, consider the three reaction mechanisms (catalysis, degradation, and annihilation) as follows:

(2) $$u^{\pm }\to u^{\pm }+y^{\pm },$$

(3) $$u^{\pm }\to \phi ,$$

(4) $$y^{+}+y^{-}\to \phi ,$$

where $u^{+}$, $u^{-}$, $y^{+}$, and $y^{-}$ are the concentrations of the chemical species $U^{+}$, $U^{-}$, $Y^{+}$, and $Y^{-}$, respectively. The symbol ϕ denotes that the concentration of the signal is zero. The formalism $u^{\pm }\to u^{\pm }+y^{\pm }$ denotes the abbreviation of the two reaction mechanisms of $u^{+}\to u^{+}+y^{+}$ and $u^{-}\to u^{-}+y^{-}$, and the directing arrow indicates a biomolecular reaction. In the design method using a linear I/O system [12], a desired circuit can be rationally constructed by using catalysis, degradation, and annihilation as the fundamental mechanism. In this paper, we call this method “pseudo-linearization”.

### 3.2. Design of Molecular PI Controller

PI control is a typical method to achieve regulation control and is the most widely used control law in the industry. PI controller is constructed with gain, integrator, adder, and subtractor circuits (see [12] for details). First, we review how to construct these basic circuits of the PI controller. The integrator with input *u* and output *y* can be designed by connecting the catalysis and annihilation reactions in series as follows:

(5) $$u^{\pm }\overset{\alpha }{\to }u^{\pm }+y^{\pm },$$

(6) $$y^{+}+y^{-}\overset{\mu }{\to }\phi ,$$

where α and μ are the reaction rates. Then, based on the reaction kinetics, the corresponding ordinary differential equations are given by the following equations:

(7) $$\begin{array}{c} {\dot{u}}^{+}={\dot{u}}^{-}=0. \end{array}$$

(8) $$\begin{array}{c} {\dot{y}}^{+}=\alpha u^{+}-\mu y^{+}y^{-}, \end{array}$$

(9) $$\begin{array}{c} {\dot{y}}^{-}=\alpha u^{-}-\mu y^{-}y^{+}, \end{array}$$

By subtracting Equation (9) from Equation (8), we obtain the following equation:

(10) $$\begin{array}{c} \dot{y}={\dot{y}}^{+}-{\dot{y}}^{-}=\alpha u, \end{array}$$

Performing a Laplace transformation, leads to the following equation:

(11) $$\begin{array}{ccc} & \frac{Y(s)}{U(s)}=\frac{\alpha }{s}, & \end{array}$$

which represents the transfer function of an integrator with the gain α. The adder with two inputs $u_{i}\;\left(i=1,2\right)$ and output *y* is created by catalysis, degradation, and annihilation reactions as follows:

(12) $$u_{i}^{\pm }\overset{\gamma k_{i}}{\to }u_{i}^{\pm }+y^{\pm },$$

(13) $$y^{\pm }\overset{\gamma }{\to }\phi ,$$

(14) $$y^{+}+y^{-}\overset{\mu }{\to }\phi ,$$

where $k_{i}\;\left(i=1,2\right)$, γ, and μ are the reaction rates. As with the integrator, we can formulate the differential equation in the form of a Laplace transformation and is given by the following equation:

(15) $$\begin{array}{ccc} & Y(s)=\frac{\gamma }{s+\gamma }\sum_{i=1}^{n}k_{i}U_{i}\left(s\right), & \end{array}$$

which represents the transfer function of the adder. Increasing the reaction rate γ in Equation (15) makes it more ideal for the adder. Note that this adder can be used as an additional point with weights (gain) by adjusting $k_{1}$ and $k_{2}$.

**Figure 1:**
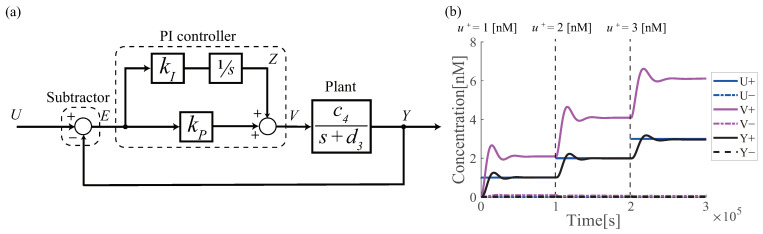
(**a**) Block diagram of the PI controller. This controller consists of 162 DNA strands. (**b**) simulation results of the PI controller. Each reaction rate is $c_{1}=c_{2}=c_{3}=k_{I}=k_{P}=0.0008$, $c_{4}=0.0004$, and $k=1.0\times 10^{-3}$ ${\mathrm{nM}}^{-1}s^{-1}$. The concentrations of fuel DNA strands used in the PI controller were obtained from the program (Examples—PI Controller—2Domain) on Visual DSD [26] and are shown in Figure S1. The input $U^{+}$ is first given 1 nM at 0 s, then the input is increased to 2 nM at $1.0\times 10^{5}$ s, and then to 3 nM at $2.0\times 10^{5}$ s.

Figure 1a shows the block diagram of the whole system. Based on the above results, the subtractor and PI controller can be realized by integrating the integrator (5)∼(6) and adder reactions (12)∼(14), where the proportional gain $k_{p}$ is implicitly included in the reaction process of the adder. The controlled plant is given by the 1st order lag element as a toy example. The corresponding ordinary differential equations of the whole system is summarized as follows:

Subtractor:

(16) $$\begin{array}{c} u^{\pm }\overset{c_{1}k}{\to }u^{\pm }+e^{\pm }, \end{array}$$

(17) $$\begin{array}{c} y^{\pm }\overset{c_{2}k}{\to }y^{\pm }+e^{\pm }, \end{array}$$

(18) $$\begin{array}{c} e^{+}+e^{-}\overset{}{\to }\phi , \end{array}$$

(19) $$\begin{array}{c} e^{\pm }\overset{d_{1}k}{\to }\phi , \end{array}$$

PI Controller:

(20) $$\begin{array}{c} e^{\pm }\overset{k_{I}k}{\to }e^{\pm }+z^{\pm }, \end{array}$$

(21) $$\begin{array}{c} e^{\pm }\overset{k_{P}k}{\to }e^{\pm }+v^{\pm }, \end{array}$$

(22) $$\begin{array}{c} z^{\pm }\overset{c_{3}k}{\to }z^{\pm }+v^{\pm }, \end{array}$$

(23) $$\begin{array}{c} z^{+}+z^{-}\overset{}{\to }\phi , \end{array}$$

(24) $$\begin{array}{c} v^{+}+v^{-}\overset{}{\to }\phi , \end{array}$$

(25) $$\begin{array}{c} v^{\pm }\overset{d_{2}k}{\to }\phi , \end{array}$$

Plant:

(26) $$\begin{array}{c} v^{\pm }\overset{c_{4}k}{\to }v^{\pm }+y, \end{array}$$

(27) $$\begin{array}{c} y^{\pm }\overset{d_{3}k}{\to }\phi , \end{array}$$

(28) $$\begin{array}{c} y^{+}+y^{-}\overset{}{\to }\phi , \end{array}$$

Figure 1b demonstrates the simulation results of the whole system, indicating that the regulation of the output *Y* to the reference concentration is successfully achieved.

**Remark** **1.** *Since the purpose of this study is to show in principle that by making the individual modules renewable, the entire DNA circuit constructed by combining them can also become renewable, unintended reactions, such as leaks, were not considered in all simulations.*

## 4. Problem Statement

DNA circuits are driven by the administration of “input” strands while consuming “fuel” strands that have initial concentrations. Because the concentrations of fuel strands gradually decrease as the reactions progress, the performance of the DNA circuit is also degraded over time. This performance degradation is an important problem in DNA circuits that achieve the desired performance requirements in dynamic equilibrium conditions, such as feedback regulators [17].

**Figure 2:**
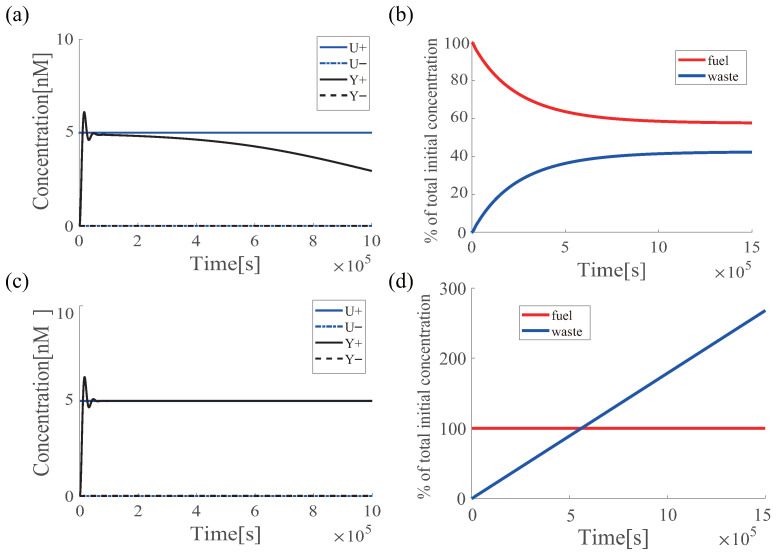
Performance degradation of the DNA-PI controller. All reaction rates are the same as those in Figure 1. Only the input $U^{+}$ was changed to 5 nM. (**a**) Time-course change in concentration of input $U^{\pm }$ (blue line) and output $Y^{\pm }$ (black line); (**b**) time-course change in total concentration of fuel (red line) and waste (blue line) strands; (**c**) time-course change in concentration of input $U^{\pm }$ (blue line) and output $Y^{\pm }$ (black line) when fuel strands are not consumed; and (**d**) time-course change in total concentration of fuel (red line) and waste (blue line) strands when fuel strands are not consumed.

When the PI controller described in Section 3.2 is given 5 nM of input $U^{+}$ as a target concentration and driven for $1.0\times 10^{5}$ s, the output $Y^{+}$ tends to follow the target concentration initially (e.g., <$2\times 10^{5}$ s), but it gradually moves away from the target concentration over time (Figure 2a), which is known to be “finite-time regulation” [16]. The total amount of fuel strands existing in the reaction system continues to decrease (Figure 2b), which is the main contributor to undermining the tracking capability. In a hypothetical simulation setting in which fuel strand consumption does not occur (Figure 2d: red line), the regulation performance is maintained during the simulation period (Figure 2c).

To drive the DNA circuit for a long time, fuel strands should be regenerated to the initial concentrations, which corresponds to making the DNA reaction “renewable”. A renewable DNA reaction can be defined as follows:

Consider the DNA circuit described by the following equation:

(29) $$\dot{x}=f\left(x\right),\;x\left(0\right)=x_{0},$$

where $x={\left[x_{1}\;x_{2}\;\cdots \;x_{n}\right]}^{T}\in R^{n}$ denotes the state vector of which element $x_{i}$ (i=1,…,n) is the concentration of single or double-stranded DNA, and $f:R^{n}\to R^{n}$ is a nonlinear function characterized by the mass action law. Since the renewability of DNA circuits can be characterized by DNA strands whose initial concentrations are given, we introduce the concept of “total fuel quantity” as follows:

**Definition** **1** (total fuel quantity and total waste quantity). *For the DNA circuit (29), let $I_{\mathrm{fuel}}$ and $I_{\mathrm{waste}}$ be index sets such that*

(30) $$\begin{array}{ccc} I_{\mathrm{fuel}} & = & \{i\in R\;|\;x_{i}\left(0\right)>0,\;i=1,\ldots ,n\}, \end{array}$$

(31) $$\begin{array}{ccc} I_{\mathrm{waste}} & = & \{i\in R\;|\;x_{i}\left(0\right)=0,\;i=1,\ldots ,n\}, \end{array}$$

*where $I_{\mathrm{fuel}}⋃I_{\mathrm{waste}}=\left\{1,\ldots ,n\right\}$ and $I_{\mathrm{fuel}}⋂I_{\mathrm{waste}}=\phi$. Then, “total fuel quantity” $T_{f}:R\to R$ and “total waste quantity” $T_{w}:R\to R$ are defined as follows:*

(32) $$\begin{array}{ccc} T_{f}\left(t\right) & = & \sum_{i\in I_{\mathrm{fuel}}}x_{i}\left(t\right), \end{array}$$

(33) $$\begin{array}{ccc} T_{w}\left(t\right) & = & \sum_{i\in I_{\mathrm{waste}}}x_{i}\left(t\right). \end{array}$$

**Remark** **2.** *As the dual concept of the total fuel quantity, we also describe the total waste quantity.*

We then create the following definition of renewable DNA circuits.

**Definition** **2** (renewable DNA circuit). *For the DNA circuit (29), let $x(t;x_{0})$ be a solution of (29) at time t starting from $x_{0}$. If, for any given $t_{r}>0$, there exists a reaction mechanism $g:R^{n}\to R^{n}$ such that for $t\geq t_{r}$,*

(34) $$\dot{\bar{x}}=g\left(\bar{x}\right),\;\bar{x}\left(t_{r}\right)=x\left(t_{r};x_{0}\right),$$

*and the time $t_{1}>t_{r}$ such that*

(35) $${\bar{T}}_{f}\left(t\right)>T_{f}\left(t_{r}\right),\;\forall t\geq t_{1},$$

*where ${\bar{T}}_{f}:R\to R$ is the total fuel quantity of (34), calculated using the following equation:*

(36) $$\begin{array}{ccc} {\bar{T}}_{f}\left(t\right) & = & \sum_{i\in I_{\mathrm{fuel}}}{\bar{x}}_{i}\left(t\right),\;t\geq t_{r}. \end{array}$$

*Then, the DNA circuit is considered “renewable” with respect to $x_{0}$. Moreover, for a given time $t_{e}>t_{1}$, which is typically the end time of the regeneration process, the efficiency of renewability is defined as follows:*

(37) $$r_{e}=1-\frac{T_{f}\left(0\right)-{\bar{T}}_{f}\left(t_{e}\right)}{T_{f}\left(0\right)-T_{f}\left(t_{r}\right)}.$$

**Remark** **3.** *The positive constant $r_{e}$ represents the error between the initial and regenerated concentrations. Note that this definition only specifies the regeneration of initial concentrations, not the circuit performance. To determine the constant $r_{e}$, the recovery of circuit performance should be evaluated in each application as discussed in Section 6.*

**Remark** **4.** *Equation (37) can be rewritten in $T_{w}$ about the efficiency of renewability.*

In summary, in this study, a renewable PI controller was designed to overcome the finite-time regulation problem of feedback regulators.

## 5. Previous Works

### 5.1. Photoisomerization of Azobenzene

**Figure 3:**
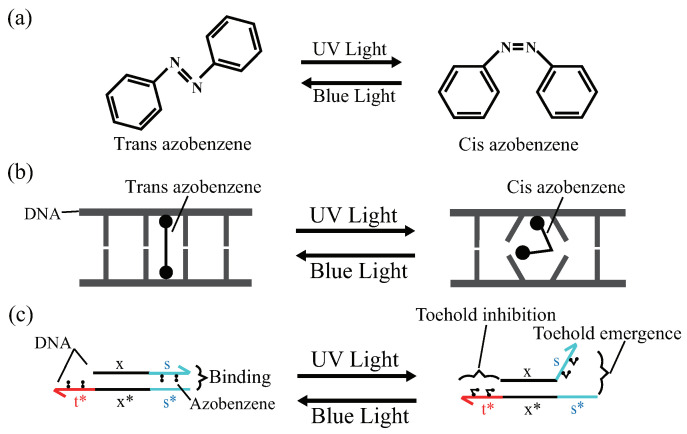
Photoisomerization of azobenzene and the effect of attachment to DNA. (**a**) Photoisomerization of azobenzene; (**b**) effect of azobenzene attachment on base pairing; and (**c**) effect of azobenzene attachment on DNA domain. The black line, x, indicates a long domain (recognition domain) of more than 15 nt, while the non-black-colored lines, t, and s, indicate short domains (toehold domain) of 1∼10 nt. In addition, x and $x^{*}$ and s and $s^{*}$ represent complementary DNAs, respectively.

To make the PI controller renewable, a renewable design of a DNA reaction system using the photoresponsive molecule, azobenzene, proposed by Song et al. [23] was applied (Song et al. made the seesaw gate renewable). Azobenzene has two structures (trans and cis forms) that can be converted into cis and trans forms under ultraviolet (UV) and blue light (BL) irradiation, respectively (Figure 3a) [22]. By applying azobenzene modification within the DNA base sequence, the stability of the double-helix structure can be controlled by light irradiation; the trans form under BL irradiation stabilizes the double-stranded structure, whereas the cis form under UV irradiation destabilizes the double-stranded structure (Figure 3b). This basic property of azobenzene is applied to DNA strand displacement reactions. By applying azobenzene modification to the toehold domain, it is possible to control the flow of the reaction process by switching the binding affinity of the toehold domain by light irradiation (Figure 3c). In “toehold inhibition”, the number of bases in the toehold is decreased by sequestering the toehold with azobenzene. In “toehold emergence”, the number of bases in the toehold is increased by tearing off the double-stranded structure.

### 5.2. Renewable Design of Three Basic Circuits

**Figure 4:**
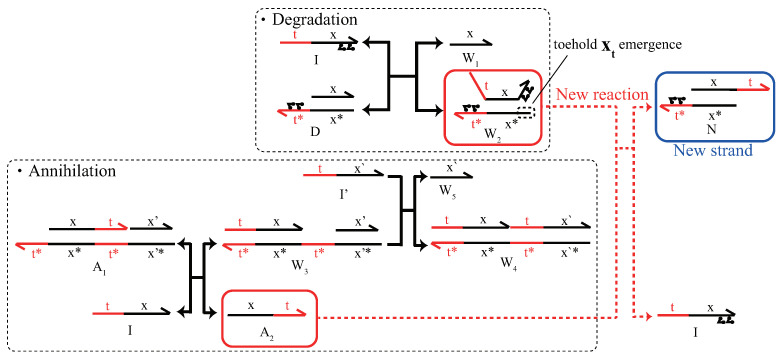
Unexpected reaction of azobenzene on annihilation and degradation circuits.

The renewable design was applied to the degradation reaction as an example. Figure 4 shows the degradation circuit with azobenzene attached and the original annihilation circuits. Briefly, the degradation circuit generates the output strand $W_{1}$ (single-stranded DNA) and the waste strand $W_{2}$ (double-stranded DNA) upon input *I*, wherein the azobenzene-modified gate *D* is prepared. The annihilation circuit annihilates two inputs *I* and $I^{\prime }$ through the two DNA strand displacement reactions while generating the waste strands $A_{2}$, $W_{3}$, $W_{4}$, and $W_{5}$. In this case, it is necessary to attach azobenzene to the recognition domain x. Then, azobenzene causes a toehold $x_{t}$ to appear and react with strand $A_{2}$, to create a new strand *N* that cannot revert to the original strand.

**Figure 5:**
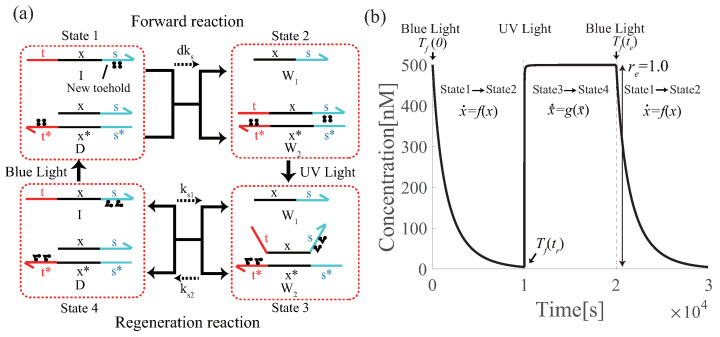
(**a**) Azo-degradation circuit; and (**b**) simulation results of input *I* for azo-degradation. The concentrations of *D* and *I* is 1000 nM and 500 nM, respectively. The binding strength *d* is 0.0008, and the reaction rate $k_{s}=1.0\times 10^{-3}\;{\mathrm{nM}}^{-1}s^{-1}$. The toehold length is 5 nt, the length of the recognition domain is 15 nt, and the concentration of the initial fuel strand (*D*) is 1000 nM. The specific reaction rates are listed in Table 1.

**Figure A1:**
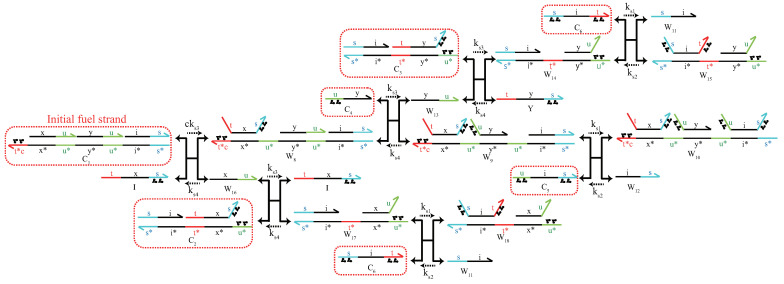
Catalysis circuit with azobenzene attached.

**Figure A2:**
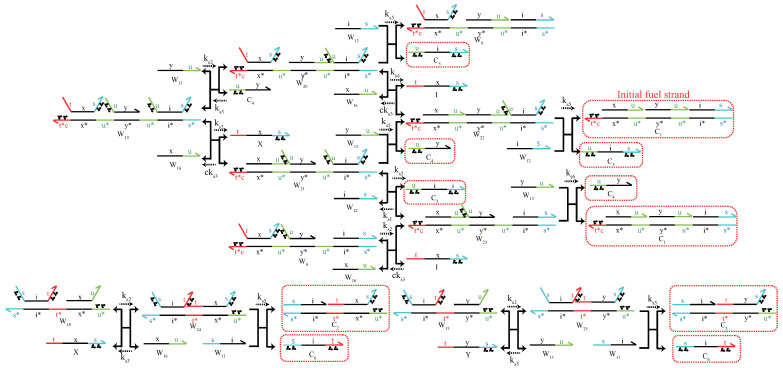
New reaction in catalysis circuits caused by azobenzene attachment.

**Figure A3:**
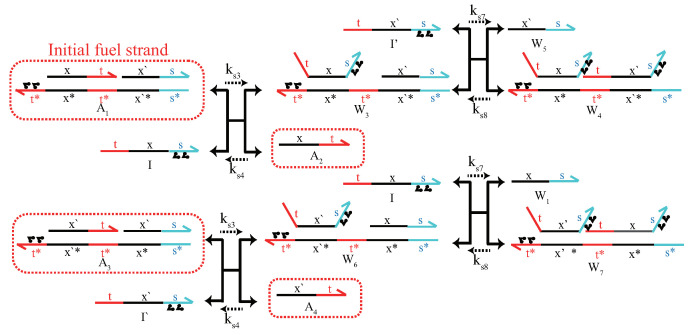
Annihilation circuit with azobenzene attached.

To overcome this problem, domain s is added to some DNAs [24] in contrast to the two-domain implementation by the pseudo linearization method proposed in [14]. The reaction scheme and simulation results of the degradation circuit with azobenzene modifications (hereafter, referred to as azo-degradation circuits) are presented in Figure 5. First, when azobenzene is in the trans form, the free energy of state 2 in Figure 5a is smaller than that of state 1 in the degradation circuit because of the difference in the member of hydrogen binding relating to the toehold $t^{*}$. As a result, the forward reaction becomes dominant. Upon irradiation with UV light, azobenzene transforms into the cis form, and the state shifts from state 2 to state 3. Then, in state 3, the toehold s is exposed owing to the destabilization of the hybridization by azobenzene, and the free energy increases, whereas in state 4, free energy decreases owing to the sealing of $t^{*}$. Therefore, the state shifts from a high free-energy state 3 to a low free-energy state 4. Then, upon irradiation with blue light, the state returns from state 4 to state 1, and the circuit returns to its original state. As a result, the concentration is completely recovered, which satisfies Definition 2 with $r_{e}=1.0$. Other renewable circuits for the annihilation and catalysis circuits are shown in Figure A1, Figure A2 and Figure A3.

## 6. Results & Discussions

### 6.1. Renewable Design of PI Controller

To make the PI controller renewable, azobenzene was added to the three basic circuits (Figure A1: Catalysis, Figure A2: Annihilation, and Figure 5a: Degradation). The PI controller was designed by combining these three circuits; i.e., Equation (16)–(28). At this time, new reactions are generated during catalysis (Figure A2), but they are eventually regenerated into fuel DNA strands. All reactions of the PI controller in the trans form of azobenzene are shown in Figures S2 and S3. The fuel DNA concentrations used in the PI controller were obtained from a program on Visual DSD [26] and are presented in Figure S1.

**Figure 6:**
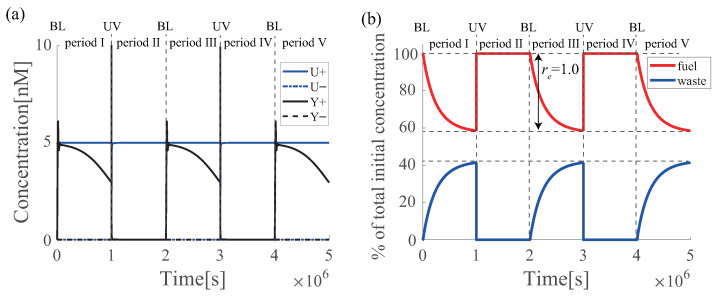
Simulation results of the renewable azo-PI controller. (**a**) Renewable performance of azo-PI controller; and (**b**) ratio of fuel DNA and waste DNA to the total initial concentration of the azo-PI controller. Since, the azo-PI controller appears in the reutilization (Figure A2), thus, the azo-PI controller consists of 220 DNA strands.

The simulation results of the renewable azo-PI controller are shown in Figure 6, where UV and BL are alternately irradiated at regular intervals, which are designated as I∼V, respectively. In period I, azobenzene exists in the trans form, and the DNA circuit functions as a PI controller, although the regulation performance of the output $Y^{+}$ to the target $U^{+}$ gradually degrades (Figure 6a) as the fuel strands decrease with time (Figure 6b). The concentration is recovered in period II by applying UV to make it cis-azobenzene, and each DNA strand returns to its initial state. Then, with the BL irradiation at III, the output *Y* with the same trajectory as that from period I appear. Figure 6b shows the ratio of fuel DNA and waste DNA to the total initial concentration. Fuel DNA decreased with time, whereas waste DNA increased as the fuel DNA decreased. UV irradiation in period IV restored the concentration to the initial state while waste DNA decreased. The concentration is completely recovered and $r_{e}=1.0$, which satisfies Definition 2. Because the azo-PI controller follows Definitions 2, the PI controller becomes renewable owing to the attachment of azobenzene. In addition, the azo-PI controller can be reused many times by using azobenzene because the circuit can be regenerated by UV again in period IV.

### 6.2. Effectiveness of Azobenzene

The simulation result of the PI controller shows the ideal case of the effectiveness of azobenzene. However, only four or three bases may be peeled off, even when five bases are required because of temperature-, buffer-, and sequence-dependent factors [22,27]. Following the results of previous studies [23,24], we defined this uncertainty as to the toehold length ($n_{s}$). The effect of azobenzene on the total length of the toehold ($n_{f}$) was defined separately as toehold initiation and toehold emergence and is expressed as the effectiveness of azobenzene (%).

(38) $$Toehold\;inhibition:\frac{n_{f}-n_{s}}{n_{f}},$$

(39) $$Toehold\;emergence:\frac{n_{s}}{n_{f}},$$

For example, when the toehold is five bases and the effectiveness of azobenzene is 60%, the inhibition is (5−2)/5, decreasing from 5 to 2 nt, and the emergence is 3/5, increasing from 0 to 3 nt.

**Figure 7:**
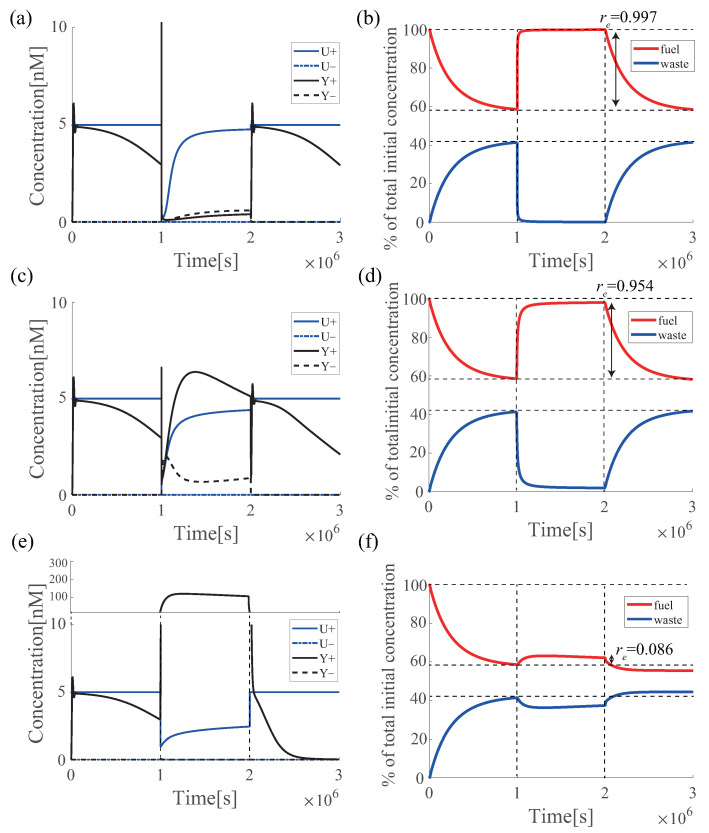
Performance and fuel DNA renewable of azo-PI controller at each effectiveness value of azobenzene. (**a**) Simulation result of performance renewable in input U (blue line) and output Y (black line); (**b**) total concentration of fuel(red) and waste (blue) strands when the effectiveness of azobenzene is 60%; (**c**) simulation result of azo-PI controller in input U (blue line) and output Y (black line); (**d**) total concentration of fuel(red) and waste (blue) strands when the effectiveness of azobenzene is 40%; (**e**) simulation result of azo-PI controller in input U (blue line) and output Y (black line); and (**f**) total concentration of fuel (red) and waste (blue) strands when the effectiveness of azobenzene is 20%. The reaction rates are listed in Table 1.

**Figure 8:**
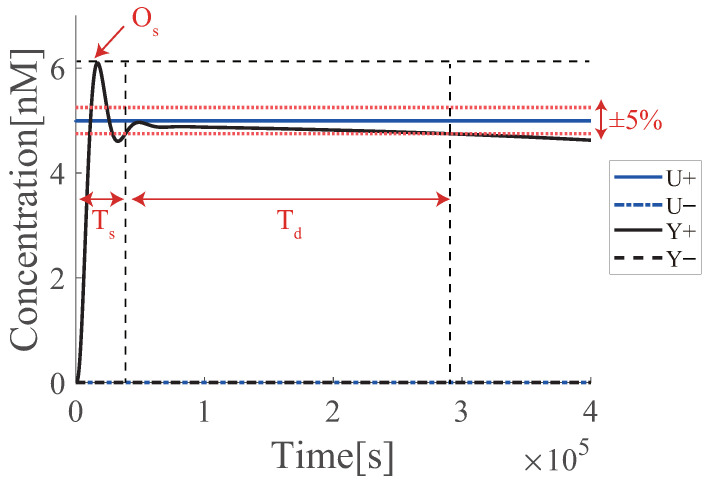
Performance indicators of DNA-PI controller.

Figure 7 shows the result of each simulation (also see Section 6.4 for the experimental evaluations). Figure 7a,b show the results of the azo-PI controller when the effectiveness of azobenzene is 60%; c and d at an effectiveness of 40%; and e and f at an effectiveness of 20%. For each effectiveness value, the performance was compared with $r_{e}$ of fuel DNA and overshoot ($O_{s}$), settling time ($T_{s}$), and duration time ($T_{d}$) of the target tracking of the PI controller (Figure 8). The results are presented in Table 3. Note that the settling time was set for the sake of convenience because the system enters the acceptable range (in this case, ±5%) and then exits the range at time $T_{d}$.

The $r_{e}$ value in Table 3 shows that the concentration is renewable for all effectiveness values of azobenzene from 60% to 20%. However, the concentration is only slightly recovered at 20%, $r_{e}=0.086$, whereas it is renewable at 60%, $r_{e}=0.997$. The PI controller’s performance index indicates that the performance at 60% is almost the same for the second time, whereas the 20% effectiveness has a very short $T_{d}$ and is not able to track the output. Therefore, at 20%, fuel DNA is renewable, but the performance of the PI controller is not renewable. At 40%, fuel DNA is sufficiently regenerated with $r_{e}=0.954$, although the performance is slightly degraded owing to the shortening of $T_{s}.$ Therefore, 40% is considered to be the limit where both, the fuel DNA and circuit performance of this PI controller, can be made renewable. Therefore, a very high $r_{e}$ is required to make the performance renewable. Note that e varies depending on the design parameters of the controller, such as the initial fuel concentration and the number of toeholds.

### 6.3. Proof-of-Principle of Renewable Design

**Figure 9:**
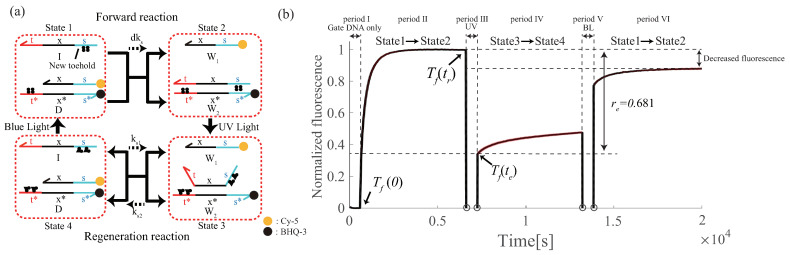
(**a**) Azo-degradation used in the experiment; and (**b**) the results of the renewable experiment of the azo-degradation circuit. This result is the average of three times with 500 nM input, 1000 nM gate, and 37.5 ${}^{\circ }$C. Colored areas represent the standard deviations.

We experimentally investigated the feasibility of our renewable design using a photoresponsive control method. To reduce the occurrence of unintended reactions, such as leaks originating from azobenzene modifications, in the DNA circuit, a simpler design should be employed. Therefore, we focused on the proof-of-principle study with the azo-degradation circuit shown in Figure 9a, where the state transitions around four states (State 1 > State 2 > State 3 > State 4) are confirmed by alternating UV and BL irradiation. The base sequences of the input strand *I* and the gate strand *D* are shown in Table 1. Azobenzene was modified at six and three positions (denoted by X in Table 1) for domain s of *I*, domain s of the upper *D*, and domain t of the lower *D*, respectively. The experimental results are presented in Figure 9b, where the fluorescence intensities were normalized between 0 and 1, using the minimum and maximum values of the measured data. For convenience, we divided the experiment duration into six periods (I, II, III, IV, V, and VI).

Period I: Without the administration of the input strand *I*, the fluorescence intensities were not detected because the lower strand of gate *D* was modified with quenching molecules and the upper strand with fluorescent molecules.

Period II: Upon administration of the input strand *I*, the fluorescence intensities increased, which indicated that the output $W_{1}$ labeled by Cy5 was released, and therefore, the transition was made from state 1 to state 2.

Period III: To photoisomerize trans-azobenzene to cis-azobenzene, the cuvette (sample) was removed from the fluorescence spectrophotometer, irradiated with UV (365 nm) for 10 min outside the instrument, and then returned to the spectrophotometer in preparation for the measurement during the next period. Because irradiation for a few minutes is sufficient for photoisomerization from the trans- to cis-form of azobenzene according to [22], this irradiation time is feasible. Note that no measurements were taken during this period because, in principle, it is difficult to precisely measure fluorescence intensity during UV irradiation for photoisomerization, and it was not possible to observe the state transition from state 2 to state 3 in this experiment.

Period IV: Under UV irradiation, in addition to the transition from state 2 to state 3, the transition from state 3 to state 4 also proceeds simultaneously. Under these circumstances, the single-stranded output $W_{1}$ is returned to gate *D*, which means that the fluorescence tends to be quenched again. As observed in the response, the signal intensity at the beginning of period IV drastically decreased. Moreover, a gradual increase in intensity was observed during this period. Although the detailed mechanism should be further investigated, we can hypothesize that the transition from state 1 to state 2 also occurred at the same time because the cis-form azobenzene tended to return to the trans form without UV irradiation.

Period V: Similar to the procedure in Period III, to photoisomerize cis-azobenzene to trans-azobenzene, the cuvette (sample) was removed from the fluorescence spectrophotometer, irradiated with BL (465 nm) for 10 min outside the instrument, and then returned to the spectrophotometer in preparation for the measurement during the next period. Note that no measurements were taken during this period.

Period VI: Similar to the circumstances in Period IV, under BL irradiation, in addition to the transition from state 4 to state 1, the transition from state 1 to state 2 also proceeds simultaneously in principle. Then, the output $W_{1}$ is released from gate *D*, which means that the fluorescence tends to increase and can be as large as the peak value observed in Period II. As observed in the response, signal intensity at the beginning of period VI drastically increased, followed by a gradual increase. In contrast, the second output after the renewable process during Period IV was smaller than that of the first output during Period II. The details of this mechanism should also be investigated for two main reasons: (i) the destruction of fluorescent molecules by UV irradiation, and (ii) the insufficient isomerization of azobenzene to the trans form by BL irradiation because perfect photoisomerization with 100% efficiency is not possible. Estimation of the efficiency of renewability: Based on Definition 2, the efficiency of renewability can be evaluated by calculating the parameter $r_{e}$. $r_{e}=0.681$ satisfies Definition 2; thus, the azo-degradation circuit is considered renewable.

### 6.4. Estimation of the Effectiveness of Azobenzene under UV Irradiation

**Figure 10:**
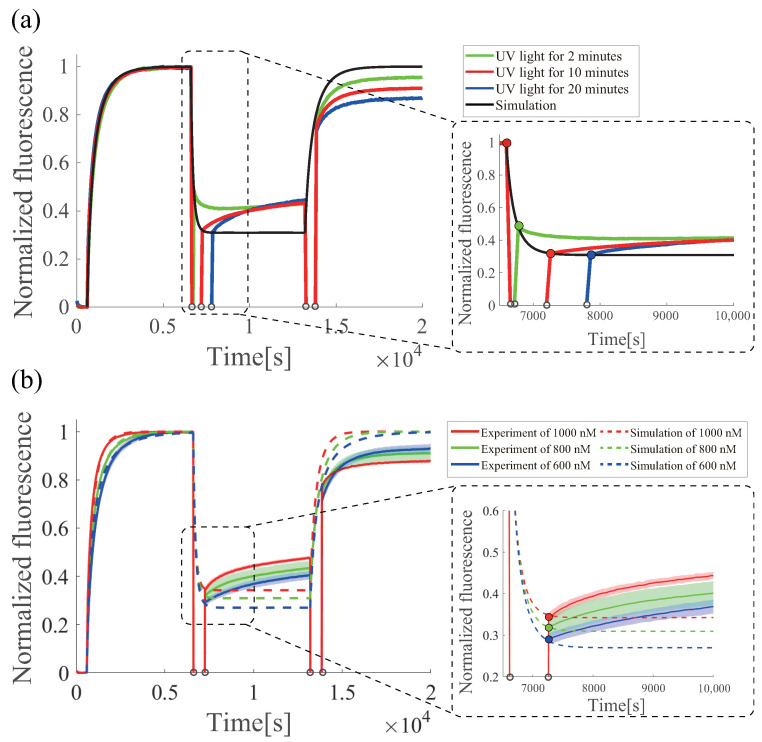
Parameter fitting of azo-degradation experiments and simulations. (**a**) Fitting at different UV irradiation times; and (**b**) fitting at different gate concentrations. Different gate concentrations were averaged over three times, and the UV irradiation time experiments were conducted once. Colored areas represent the standard deviations.

By comparing the experimental and simulation data of the azo-degradation circuit, we can evaluate the effect of azobenzene on the “toehold inhibition” and “toehold emergence” under photoresponsive control. For this purpose, we attempted to estimate the reaction rates of the azo degradation circuit based on experimental data. Although time-course data are needed to estimate the parameters of the ordinary differential equation model, no measurements can be taken during the UV irradiation period (Period III). Hence, the data from Period IV should be employed for parameter estimation. However, a gradual increase was also observed during Period IV after UV irradiation, which may be caused by complex phenomena, including the reaction regarding the state transition from state 1 to state 2. Therefore, to perform valid parameter estimation, only the first time-point data of Period IV were employed. Moreover, to collect the time-series data required for parameter estimation, experiments with different UV irradiation periods (2, 10, and 20 min) in Period III were conducted (Figure 10a), where the initial concentrations of gate *D* and input *I* were 800 nM and 500 nM, respectively. Figure 10a shows that the renewable reaction (state 3 to state 4) was not completed in 2 min but almost completed in 10 min because there was no difference in the fluorescence level between 10 and 20 min. By using the four data points from (i) the last time-point of Period II, (ii) the first time point of Period IV in the case of 2 min UV irradiation, (iii) the first time point of Period IV in the case of 10 min UV irradiation, and (iv) the first time point of Period IV in the case of 20 min UV irradiation, we estimated the simulation result based on the ordinary differential equation of azo-degradation with the estimated parameters $dk_{s}$, $k_{s1}$, and $k_{s2}$ as illustrated by the black solid line in Figure 10a. The reaction rate in the forward direction was $dk_{s}=4.0\times 10^{-6}$ ${\mathrm{nM}}^{-1}s^{-1}$, and in the reverse direction, the fitted values $k_{s1}=1.45\times 10^{-6}$ ${\mathrm{nM}}^{-1}s^{-1}$ and $k_{s2}=1.35\times 10^{-5}$ ${\mathrm{nM}}^{-1}s^{-1}$ were in good agreement. To validate the fitting parameters, the fluorescence intensities were measured at different initial concentrations of D(0)=600,800,1000 nM (Figure 10b), where input was I(0)=500 nM and UV irradiation period was 10 min. As a result, the reaction rate in the forward direction was $dk_{s}=4.0\times 10^{-6}$ ${\mathrm{nM}}^{-1}s^{-1}$ at 1000 and 800 nM and $dk_{s}=6.0\times 10^{-6}$ ${\mathrm{nM}}^{-1}s^{-1}$ at 600 nM. In the reverse direction, the fitted values $k_{s1}=1.45\times 10^{-6}$ ${\mathrm{nM}}^{-1}s^{-1}$ and $k_{s2}=1.35\times 10^{-5}$ ${\mathrm{nM}}^{-1}s^{-1}$ were found to be in close agreement (Figure 10a).

As for the effect of azobenzene on toehold inhibition and toehold emergence, since a toehold of 3 nt results in a reaction rate of $k_{s}=4.0\times 10^{-6}$ ${\mathrm{nM}}^{-1}s^{-1}$ and a difference of one toehold results in a 10-fold change in the reaction rate, significant toeholds of the post-reaction toehold inhibition and emergence can be obtained as $3-\log\left(4.0\times 10^{-6}/1.45\times 10^{-6}\right)≓2.56$ and $4-\log\left(4.0\times 10^{-5}/1.45\times 10^{-5}\right)≓3.53$, respectively. The estimated reaction rates show that the inhibition is (3−2.56)/3=0.147 (14.7%), decreasing from 3 to 2.56 nt and the emergence is 3.53/10=0.353 (35.3%), increasing from 0 to 3.53 nt, where the yields for toehold inhibition and emergence were calculated using Equations (38)∼(39). These results suggest that the effect of toehold emergence is reasonably high, whereas the effect of toehold inhibition is low. Note that this estimation assumes that cis-azobenzene can destabilize the double-helix structure, which can be approximated by a decrease in the number of substantial base pairs, but it does not have any effect on the binding rate for hybridization.

**Figure 11:**
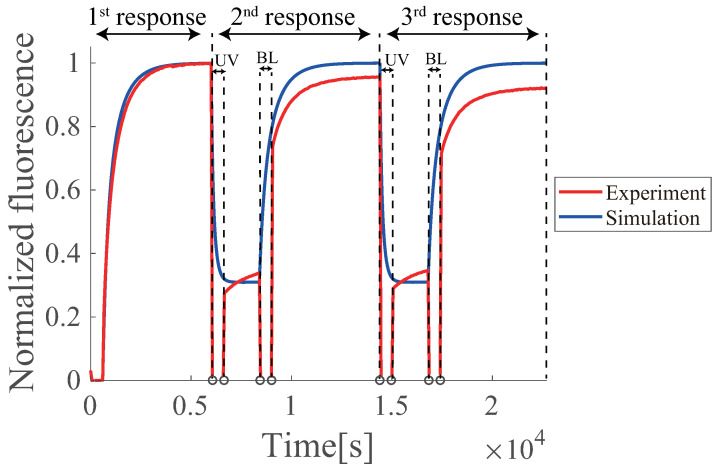
Multiple operations of azo-degradation. The red line shows the experimental results, and the blue line shows the simulation results. The simulation uses the reaction rates of $k_{s1}=1.45\times 10^{-6}$ ${\mathrm{nM}}^{-1}s^{-1}$, $k_{s2}=1.35\times 10^{-5}$ ${\mathrm{nM}}^{-1}s^{-1}$. The experiment was conducted once.

**Remark** **5.** *Figure 11 also shows that the smaller the initial concentration of fuel strand, the higher the maximum level of second output response after the renewable process.*

### 6.5. Multiple Regenerations of Azo-Degradation Circuit

To investigate multiple renewability, two consecutive regenerations were experimentally examined (Figure 11). The second and third responses appeared after the respective UV-BL irradiation period, indicating that multiple regenerations were feasible. However, unlike the ideal simulation result, the regenerated output was reduced by 5.5% on average (5% and 6% for the first and second responses, respectively). This stepwise reduction may be due to fluorescence photobleaching by UV irradiation and time elapsed, in addition to insufficient isomerization to the trans form by BL irradiation, as discussed in Section 6.3.

### 6.6. Temperature Dependence

**Figure 12:**
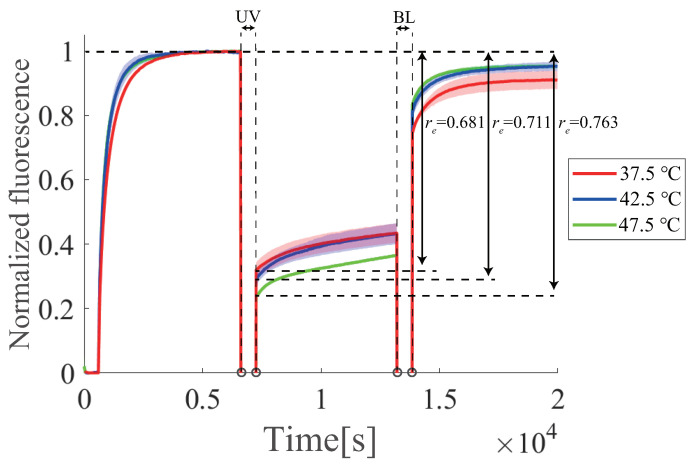
Effect of changing the temperature on azobenzene. The red line is 37.5 ${}^{\circ }$C, the blue line is 42.5 ${}^{\circ }$C, and the green line is 47.5 ${}^{\circ }$C. This result is the average of three times. Colored areas represent the standard deviations.

The temperature dependence of the regeneration efficiency $r_{e}$ was experimentally evaluated under three different temperature conditions (37.5, 42.5, and 47.5 ${}^{\circ }$C ), where the initial concentrations of gate *D* and input *I* were set to 800 nM and 500 nM, respectively. Based on Figure 12, the regeneration efficiencies were calculated to be 0.681, 0.711, and 0.763 for 37.5, 42.5, and 47.5 ${}^{\circ }$C, respectively, which indicated that the regeneration efficiency at 47.5 ${}^{\circ }$C was significantly better than those at 37.5 and 42.5 ${}^{\circ }$C, respectively. Although temperature dependence in the regeneration process was limited, it is expected that the regeneration efficiency of the circuit can be improved by optimizing the experimental temperature conditions.

### 6.7. Buffer Dependence

**Figure 13:**
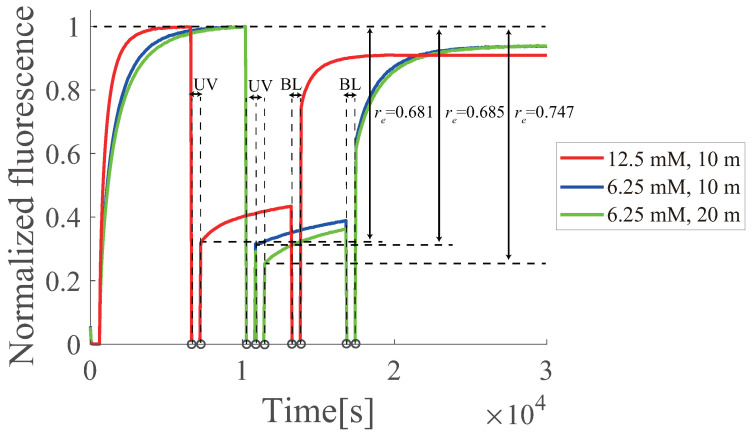
Effect of changing the buffer concentration on azobenzene. The red line shows 12.5 mM ${\mathrm{MgCl}}_{2}$, and the blue and green lines show 6.25 mM ${\mathrm{MgCl}}_{2}$. The blue line represent UV irradiation for 10 min and the green line for 20 min. The 12.5 mM experiments were averaged over three times, and the 6.25 mM experiments were conducted once.

Two different buffer conditions (TE buffer with 6.25 and 12.5 mM ${\mathrm{MgCl}}_{2}$) were examined, where the initial concentrations of the gate *D* and input *I* were 800 nM and 500 nM, respectively. Based on Figure 13, the regeneration efficiency was calculated as 0.681, 0.685, and 0.747 for ${\mathrm{MgCl}}_{2}$ concentrations of 12.5 mM (10 min UV), 6.25 mM (10 min UV), and 6.25 mM (20 min UV), respectively. In the forward reaction, the response was slower with a lower ${\mathrm{MgCl}}_{2}$ concentration (6.25 mM) because the low ${\mathrm{MgCl}}_{2}$ concentration promotes the DNA binding reaction. As for the regeneration reaction, the condition with a lower ${\mathrm{MgCl}}_{2}$ concentration and a longer UV revealed a significantly better efficiency, whereas at 12.5 mM ${\mathrm{MgCl}}_{2}$, there was no significant difference in the efficiency between UV durations of 10 and 20 min (Figure 10). In addition to temperature dependence, it is expected that the regeneration efficiency of the circuit can be improved by optimizing the buffer conditions.

## 7. Conclusions

In this study, we confirmed via simulations that the PI controller can be reused by azobenzene and the DNA circuit can be regenerated experimentally. The PI controller could regenerate the circuit up to 40% of the effectiveness of azobenzene. The PI controller may be able to improve the regeneration rate by increasing the number of bases of the added toehold and optimizing the concentration of the fuel DNA. The DNA circuit could recover the fuel DNA by reversing the reaction using azobenzene. In addition, only 60% of the fuel DNA was regenerated during the experiment. We changed the temperature and buffer concentration to increase the regeneration rate, but those changes did not significantly affect the regeneration rate. Therefore, further analyses are needed in future research, such as attaching azobenzene to the recognition domain to regenerate more fuel DNA. By contrast, since azobenzene is generally expensive (the estimated cost of the azo-PI controller is more than $10,000), further development toward cost reduction is expected, further development toward cost reduction is expected. Based on our contributions, it is expected that the “bottom-up” renewable design of combining the renewable modules can be applied to the complex information processing circuits required for molecular robots.

## Figures and Tables

**Table 1:** Reaction rates for each effectiveness value of azobenzene.

	100%	80%	60%	40%	20%	0%
$k_{s1}$	4.1410 $\times \;10^{-10}$	8.5367 $\times \;10^{-9}$	2.054 $\times \;10^{-7}$	1.7476 $\times \;10^{-6}$	2.8887 $\times \;10^{-5}$	8.5436 $\times \;10^{-4}$
$k_{s2}$	0.0011	3.6509 $\times \;10^{-5}$	2.0968 $\times \;10^{-6}$	2.3262 $\times \;10^{-7}$	9.0797 $\times \;10^{-9}$	4.1410 $\times \;10^{-10}$
$k_{s3}$	4.1410 $\times \;10^{-10}$	7.2793 $\times \;10^{-9}$	1.7928 $\times \;10^{-7}$	1.6047 $\times \;10^{-6}$	2.7996 $\times \;10^{-5}$	8.6716 $\times \;10^{-4}$
$k_{s4}$	0.0011	0.0011	0.0011	0.0010	0.0010	8.6716 $\times \;10^{-4}$
$k_{s5}$	0.0011	3.6510 $\times \;10^{-5}$	2.0976 $\times \;10^{-6}$	2.3437 $\times \;10^{-7}$	9.5163 $\times \;10^{-9}$	5.4136 $\times \;10^{-10}$
$k_{s6}$	0.0011	0.0011	0.0011	0.0011	0.0011	0.0011
$k_{s7}$	8.6716 $\times \;10^{-4}$	9.7521 $\times \;10^{-4}$	9.5901 $\times \;10^{-4}$	9.2402 $\times \;10^{-4}$	8.8792 $\times \;10^{-4}$	8.5436 $\times \;10^{-4}$
$k_{s8}$	8.6716 $\times \;10^{-4}$	2.6648 $\times \;10^{-5}$	1.4562 $\times \;10^{-6}$	1.5549 $\times \;10^{-7}$	6.0460 $\times \;10^{-9}$	3.2996 $\times \;10^{-10}$

**Table 2:** DNA used in the experiments. X refers to the modification point of azobenzene.

Sequence	Base Sequence (5${}^{\prime }$–3${}^{\prime }$)
D (under strand)	XAXGTX GGTGAGTGATGTAGG ATATAATATG—BHQ-3
D (upper strand)	Cy5—CATATTATAT CCTACATCACTCACC
I	XCXATXATXTAXTAXT CCTACATCACTCACC ACT

**Table 3:** Renewable indicators for performance of PI controller and fuel DNA.

Effectiveness of Azobenzene	$r_{e}$	$O_{s}$	$T_{s}$	$T_{d}$
100%	1.0	6.1	40,626	249,249
60%	0.997	6.1	38,639	246,655
40%	0.954	5.7	39,821	163,951
20%	0.086	107.5	39,593	16,187

## Supplementary Materials

The following are available at https://www.mdpi.com/article/10.3390/mi13020193/s1, Figure S1: Initial concentration of PI controller, Figure S2: First DNA Strand Displacement of PI controller, Figure S3: Second DNA Strand Displacement of PI controller.

## Abbreviations

The following abbreviations are used in this manuscript:

BL	Blue light
UV	ultraviolet
DNA-PI controller	PI controller designed with DNA
azo-PI controller	PI controller with azobenzene attached
azo-catalysis	catalysis with azobenzene attached
azo-degradation	degradation with azobenzene attached
azo-annihilation	annihilation with azobenzene attached

## Appendix A. Design of DNA Circuits with Attached Azobenzene

DNA circuits other than the degradation circuit described in the text and the reaction rates for each effectiveness value of azobenzene are shown in Figure A1, Figure A2 and Figure A3.
