# Supplementary material for: Renewable DNA Proportional-Integral Controller with Photoresponsive Molecules
Source: Micromachines (Basel). 2022 Jan 26;13(2):193. doi: 10.3390/mi13020193 (PMC8879760; doi:10.3390/mi13020193)
Supplement: Supplementary file 1 [file micromachines-13-00193-s001.zip › micromachines-1544023-supplementary.pdf]

Article

Renewable DNA proportional-integral controller with photoresponsive molecules

Masaaki Tamba[1], Keiji Murayama[2], Hiroyuki Asanuma[2] and Takashi Nakakuki [3]*

[1] Department of Systems Design and Informatics, Kyushu Institute of Technology, Iizuka, Fukuoka 8208502, Japan, tamba.masaaki231@mail.kyutech.jp
[2] Department of Biomolecular Engineering, Graduate School of Engineering, Nagoya University, Furo-cho, Chikusa-ku, Nagoya 4648603, Japan
[3] Department of Intelligent and Control Systems, Kyushu Institute of Technology, Iizuka, Fukuoka 8208502, Japan, nakakukil@ces.kyutech.ac.jp
* Correspondence: nakakukil@ces.kyutech.ac.jp; Tel.: +81-948-29-7716 (F.L.)

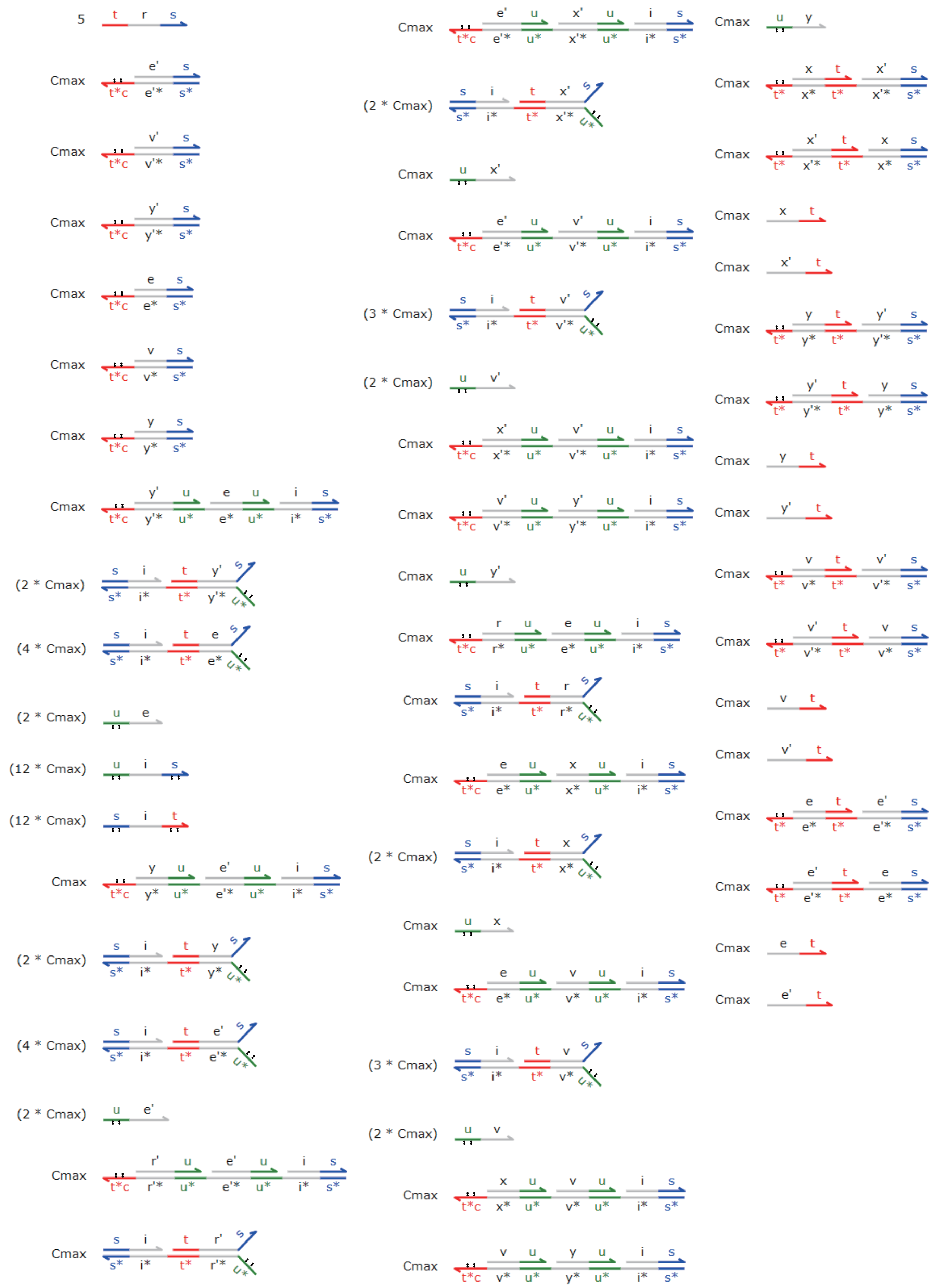

Figure S1. Initial concentration of PI controller. C_{max}=1000 nM. Domain x refers to domain z in the main text, and domain r refers to domain u in the main text.

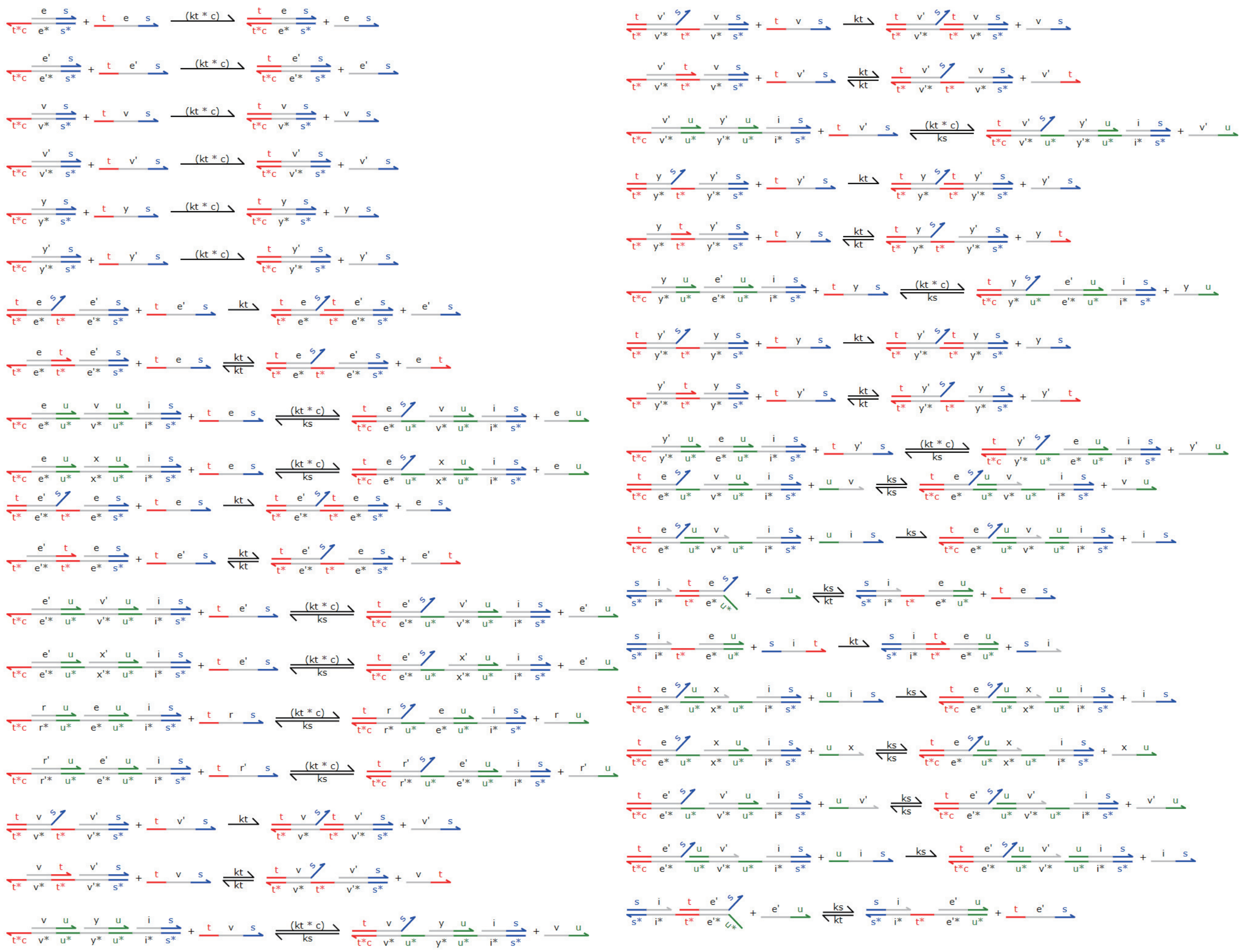

Figure S2. First DNA Strand Displacement of PI controller. Domain x refers to domain z in the main text, and domain r refers to domain u in the main text. $k_t = k_u = 1.0 \times 10^{-3}$.

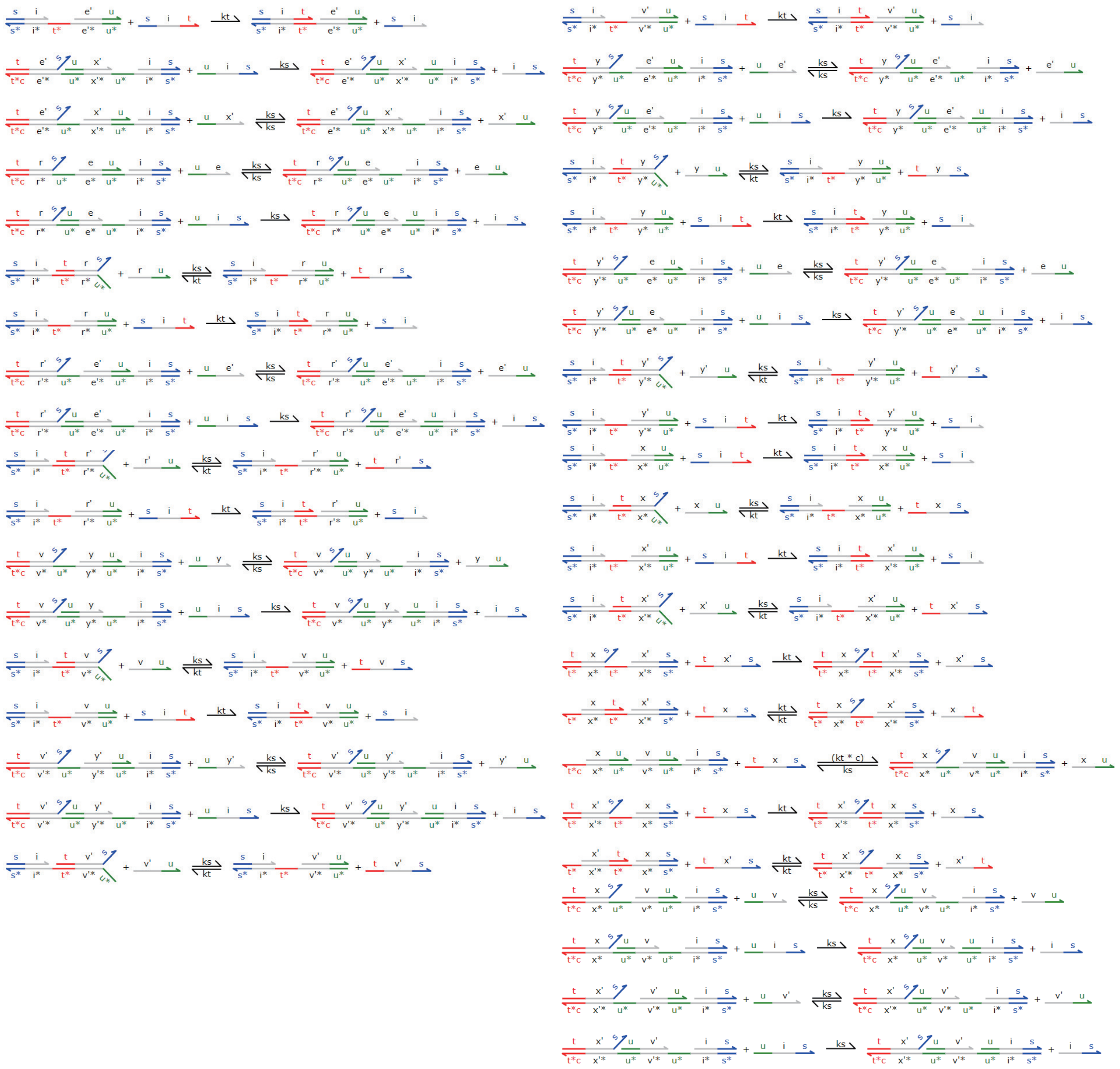

Figure S3. Second DNA Strand Displacement of PI controller. Domain x refers to domain z in the main text, and domain r refers to domain u in the main text. $k_t = k_u = 1.0 \times 10^{-3}$.
